# Nursing care for women with HELLP syndrome: a scoping review

**DOI:** 10.1590/1980-220X-REEUSP-2024-0116en

**Published:** 2024-08-12

**Authors:** Pâmela Silva Arduini, Cynthya Viana de Resende, Jéssica Aparecida da Silva, Mariana Torreglosa Ruiz

**Affiliations:** 1 Universidade Federal do Triângulo Mineiro, Uberaba, MG, Brazil.; 2 Universidade Federal do Triângulo Mineiro, Programa de Pós-Graduação em Atenção à Saúde, Uberaba, MG, Brazil.

**Keywords:** HELLP Syndrome, Nursing Care, Obstetric Nursing, Síndrome HELLP, Atención de Enfermería, Enfermería Obstétrica

## Abstract

**Objective:**

To map evidence on nursing care for women with HELLP syndrome.

**Method:**

A scoping review with searches carried out in May 2023, independently, in the PubMed/MEDLINE, LILACS, Scopus, EMBASE, Web of Science, CINAHL, CAPES Theses and Dissertations Catalog and Cochrane Library databases, correlating the descriptors HELLP Syndrome, Nursing Care and Obstetric Nursing and its synonyms, without delimitation of time and language. Selection was carried out by three researchers independently and resolved by consensus.

**Results:**

Of the 129 studies, ten were selected, which made up the final sample. The studies date from 2004 to 2022, with a predominance of English language and clinical case studies. A greater occurrence of the syndrome was observed in second-time pregnant women in the second decade of life, with a gestational age from 32 weeks, which resulted in an emergency cesarean section, and all newborns were discharged accompanied by their mothers. Studies that described nursing diagnoses and focused on nursing care were retrieved. From the review, 39 nursing care were identified.

**Conclusion:**

This review pointed out the magnitude of the syndrome, however with a lack of studies.

## RESUMEN

**Objetivo:**

Mapear evidencia sobre los cuidados de enfermería a mujeres con síndrome HELLP.

**Método:**

Revisión de alcance, con búsquedas realizadas en mayo de 2023, de forma independiente, en las bases de datos PubMed/MEDLINE, LILACS, Scopus, EMBASE, Web of Science, CINAHL, Catálogo de Tesis y Disertaciones CAPES y Cochrane Library, correlacionando los descriptores HELLP Syndrome, Nursing Care y Obstetric Nursing y sus sinónimos, sin delimitación de tiempo e idioma. La selección fue realizada por tres investigadores de forma independiente y resuelta por consenso.

**Resultados:**

De las 129 publicaciones se seleccionaron diez, que conformaron la muestra final. Las publicaciones datan de 2004 a 2022, con predominio del inglés y estudios de casos clínicos. Se observó una mayor ocurrencia del síndrome en mujeres embarazadas por segunda vez en la segunda década de la vida, con edad gestacional a partir de las 32 semanas, lo que resultó en cesárea de emergencia, y todos los recién nacidos fueron dados de alta acompañados de sus madres. Se recuperaron estudios que describían diagnósticos de enfermería y se centraban en los cuidados de enfermería. De la revisión se identificaron 39 medidas de cuidados de enfermería.

**Conclusión:**

Esta revisión señaló la magnitud del síndrome, aunque faltan estudios.

## INTRODUCTION

According to the World Health Organization (WHO), hypertensive syndromes during pregnancy account for one-tenth of maternal deaths^(1)^. On the national scene, data from the Brazilian Health System Department of Informatics (DATASUS – *Departamento de Informática do Sistema* Único *de Saúde*) indicate that, between 1996 and 2021, more than 45 thousand maternal deaths and 2,808 late deaths were recorded, totaling 48,301 maternal deaths. Of these, 62% were due to direct obstetric causes, and hypertensive syndromes accounted for 34.4% of all causes (16,622 deaths), with eclampsia being the first reason for maternal death in the country^(2)^.

A review of Brazilian studies on pre-eclampsia and eclampsia found a frequency of pre-eclampsia of 6.7%, and of eclampsia, between 1.7% and 6.2%, and HELLP syndrome represented the evolution of one in every 30 cases of pre-eclampsia, being a condition little reported, although it represents a greater severity of the evolution of the conditions^(3)^.

Pre-eclampsia is characterized by an increase in blood pressure after the twentieth week of pregnancy associated with proteinuria, which can be resolved after birth or evolve with complications and sequels. Eclampsia, on the other hand, consists of a condition that involves seizures not attributed to neurological causes in patients with a previous diagnosis of pre-eclampsia, being classified as a complication of pre-eclampsia. HELLP syndrome is a worsening of pre-eclampsia, progressing with hemolysis, elevated liver enzymes and severe thrombocytopenia, being considered the most severe evolution of gestational hypertensive syndromes^(4,5)^.

Increased blood pressure during pregnancy is considered to be values of systolic blood pressure (SBP) greater than or equal to 140 mmHg and/or diastolic blood pressure (DBP) greater than or equal to 90 mmHg, measured using an appropriate technique and confirmed by two measurements spaced four hours apart. If a SBP value greater than 160 mmHg and/or a DBP greater than 110 mmHg is obtained, a new measurement is recommended within 15 minutes and, if values are maintained, start treatment immediately. Proteinuria is laboratory confirmed by the presence of 300 mg or more of protein in 24-hour urine or a result of a urinary protein/creatinine ratio equal to or greater than 0.3, or if it is not possible to perform a 24-hour test, the presence of at least one cross in an isolated urine sample^(4,5)^.

The first description of HELLP syndrome dates back to 1982 by doctor Louis Weinstein^(6)^, who observed the emergence of symptoms from 20 weeks of gestation onwards as well as its first manifestations in the postpartum period. The syndrome’s common symptoms include sudden malaise, nausea and vomiting, and abrupt and intense abdominal pain in the right upper quadrant of the abdomen and/or epigastric quadrant, with pain being the characteristic symptom of the syndrome. Generally, the condition is associated with the complication of pre-eclampsia^(7)^; however, in 15% to 20% of cases, they may occur without hypertensive increase or without proteinuria^(8)^.

To conclude the diagnosis, laboratory tests are necessary, consisting of the following criteria: 1. Hemolysis (abnormal smear suggestive of microangiopathic hemolytic anemia characterized by the presence of schistocytes); 2. Total bilirubin above 1.2 mg/dl; 3. Lactate dehydrogenase above 6,000 U/l or hepatoglobin lower than the lower limit for normality; 4. Hepatic enzyme alanine aminotransferase with a value twice the normal limit; and 5. Platelet count less than 100,000/μl. Of these criteria, the presence of one to two suggests the possibility of HELLP, and three criteria define the diagnosis^(9)^.

Among the possible complications of the syndrome, pulmonary edema, reversible or non-reversible kidney damage, need for polytransfusions, admission to an Critical Care Unit (CCU) and disseminated intravascular coagulation are common^(8,9)^, which makes cases more serious.

HELLP syndrome is characterized as a serious and acute condition, consisting of an obstetric emergency. It affects one woman for every 45,000 live births, accounting for 0.1% to 0.9% of complications during the pregnancy-puerperal cycle, with 10% to 30% of cases presenting as a complication of pre-eclampsia^(7)^. Around 45% of HELLP cases progress seriously^(10)^. All cases require intensive care, and 1% progress to liver rupture^(11)^, a condition that poses a risk of mortality, especially when platelets reach levels below 50,000^(10,11)^.

In most cases, the etiopathogenesis is unknown. However, null or multiparity, age over 30 years, history of HELLP syndrome in a previous pregnancy, chronic hypertension, diabetes diagnosed pre-gestation, heart disease, obesity, chronic liver diseases, placental changes and congenital abnormalities are described as risk factors for HELLP syndrome^(12)^.

In addition to the negative impact and possible complications in women’s lives^(13,14)^, hypertensive syndromes in the pregnancy-puerperal period are associated with prematurity, and this is the main cause of death in children under five years of age^(15)^.

Quality prenatal care is a protective factor against complications of the syndrome, which, although it cannot be prevented, the earlier the diagnosis is made, the greater the possibility of reducing damage. Termination of pregnancy is indicated as treatment as soon as the condition is stabilized, that is, it should not be untimely or immediate, but planned to avoid negative outcomes^(5)^. Thus, due to its complexity and high potential for complications, the importance of qualified nursing care based on scientific evidence is identified.

This research aims to present a synthesis of knowledge about nursing care for women with HELLP syndrome, in a way that allows nurses to make safe and accurate decisions based on previous results described in the literature. Considering the above, this study aimed to map evidence on nursing care for women with HELLP syndrome.

## METHOD

### Study Design

This is a scoping review developed based on JBI recommendations^(16)^. In this way, the steps were taken: (1) establishment of title and review question based on the PCC mnemonic, where P: Population, C: Concept and C: Context; (2) exploration of the state of the art of the research problem with writing of review introduction; (3) inclusion criteria definition; (4) search strategy design (sources, descriptors and manual references based on reading selected studies); (5) selection of source of evidence (examiner and protocol); (6) article selection – process guided by the Preferred Reporting Items for Systematic Reviews and Meta-Analysis (PRISMA-ScR) flowchart^(17)^; (7) data extraction; (8) analysis of evidence; and (9) presentation of results in tabular form and through descriptive mapping. Protocol was registered in the Open Science Framework (https://osf.io/d8kup).

### Search Strategy

To prepare the review question, the PCC mnemonic was used, with Population (P) including pregnant or postpartum women, Concept (C) including nursing care, and Context (C) including HELLP syndrome. Therefore, the review question was: what is the evidence on nursing care for women with HELLP syndrome?

The searches were carried out in May 2023, independently, by two reviewers, a master’s student and a doctor. One reviewer has experience with search strategy and training courses for scoping reviews, and both are experts in the area of maternal and child health. The search was validated by a librarian. The descriptors HELLP Syndrome, Nursing Care and Obstetric Nursing were used. The US National Library of Medicine National Institutes of Health (MEDLINE/PubMed), Web of Science (WoS), Excerpta Medica DataBASE (EMBASE), SciVerse Scopus, Latin American and Caribbean Literature in Health Sciences (LILACS), CAPES Theses and Dissertations Catalog and Cochrane Library databases were selected for consultation, correlating the descriptors HELLP Syndrome, Nursing Care and Obstetric Nursing. No date, language and/or study design filters were applied. The search strategies are described in Chart 1, with the numerical return obtained.

**Chart 1:** Database search strategies and numerical return obtained – Uberaba, MG, Brazil, 2023.

Database	Search strategy	n
**PubMed**	(((“HELLP Syndrome” [Mesh] OR Syndrome, HELLP OR Hemolysis, Elevated Liver Enzymes, Lowered Platelets) AND (“Nursing Care”[Mesh] OR Care, Nursing OR Management, Nursing Care OR Nursing Care Management OR “Obstetric Nursing” [Mesh] OR Nursing, Obstetric OR Nursings, Obstetric OR Obstetric Nursings OR Nursing, Obstetrical OR Nursings, Obstetrical OR Obstetrical Nursings OR Obstetrical Nursing)	**25**
**EMBASE**	(‘HELLP Syndrome’/exp OR ‘HELLP (hemolysis, elevated liver enzymes and low platelets) syndrome’ OR ‘hemolysis, elevated liver enzymes, and low platelet count syndrome’ OR ‘hemolysis, elevated liver enzymes, and low platelets syndrome’ OR ‘hemolysis, elevated liver enzymes, low platelet count syndrome’ OR ‘syndrome HELLP’ OR ‘HELLP syndrome’) AND (‘Obstetrical nursing’/exp OR ‘obstetric nursing’ OR ‘obstetrics nursing’ OR ‘obstetrical nursing’ OR ‘Nursing’/exp OR ‘hospital nursing service’ OR ‘nursing service’ OR ‘nursing service, hospital’ OR ‘nursing services’ OR ‘nursing support’ OR ‘nursing, private duty’ OR ‘nursing, supervisory’ OR ‘office nursing’ OR ‘private duty nursing’ OR ‘supervisory nursing’ OR ‘nursing’) AND [embase]/lim NOT ([embase]/lim AND [medline]/lim)	**28**
**Scopus**	ALL (“HELLP Syndrome”) AND TITLE-ABS-KEY (*nursing AND care* OR *obstetric AND nursing* )	**54**
**WoS**	(ALL=(HELLP syndrome*)) AND ALL=(Nursing care*) AND (ALL=(HELLP syndrome*)) AND ALL=(obstetric nursing*)	**27**
**LILACS**	(tw:(“Síndrome HELLP” OR “HELLP Syndrome” OR “HELLP syndrome” OR C12.050.703.395.186 )) AND (tw:(“Cuidados de Enfermagem” OR “Nursing Care” OR “Atención de Enfermería” OR “Soins infirmiers” OR “Assistência de Enfermagem” OR “Atendimento de Enfermagem” OR “Cuidado de Enfermagem” OR “Gestão da Assistência de Enfermagem” OR “Sistematização da Assistência de Enfermagem” OR “E02.760.611” “OR N02.421.533” OR “Enfermagem Obstétrica” OR “Obstetric Nursing” OR “Enfermería Obstétrica” OR “Soins infirmiers em obstétrique” OR “H02.478.676.570” OR “N02.421.533.571”)) AND (db:(LILACS))	-
**CAPES**	Síndrome HELLP	**88**
**Cochrane**	HELLP syndrome	**07**

To expand the scope of the searches, terminological variations were added in different languages, synonyms for controlled descriptors and Boolean operator AND, for the simultaneous occurrence of subjects, and Boolean operator OR, for the occurrence of another subject, as summarized in Chart 1.

**Figure 1:**
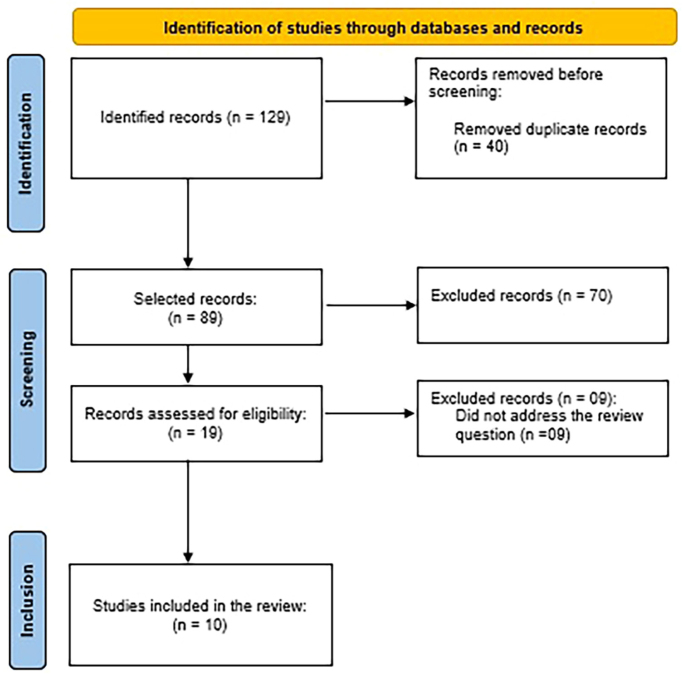
PRISMA flowchart for study selection. Uberaba, MG, Brazil, 2023. Source: prepared by the authors based on PRISMA SCr 2020^(17)^.

Studies that described nursing care for women with HELLP syndrome, without time or language limitations, were included. Duplicate articles in the databases, opinion articles, editorials, consensus(s), response letters or letters to the editor, abstracts presented in event annals and those that did not answer the review question were excluded. It is noteworthy that the level of evidence was not considered an exclusion criterion, as it is a topic little explored in the literature. PRISMA^(17)^ methodology was adopted to select articles and it was illustrated in a flowchart (Figure 1).

Study selection was carried out independently by three researchers, and disagreements were resolved by consensus, without the need to add a new reviewer at this stage. The initial analysis of selected studies was carried out by reading titles and abstracts, followed by exhaustive reading in full for the final selection of studies. Figure 1 illustrates the study selection process.

In the search, 129 studies were located. In the first stage, duplicates were removed (n = 40) and 70 articles were excluded after reading the titles and abstracts because they did not portray the study topic or did not have a suitable study design for inclusion, according to established criteria. Of the 19 studies analyzed in full, ten were selected after reading them. Thus, the final sample was composed of ten studies.

Data extraction was carried out by three researchers, independently. Standardized information was extracted by JBI, such as authorship, year, producing country, objectives, population and sample size, methodology used, outcomes, main results that answer the review question and risk of bias. The extracted data were tabulated and presented through narrative synthesis.

## RESULTS

Ten studies made up the review, published between 2004 and 2022 (30%), seven (70%) published in English and three (30%) in Portuguese. Four studies (40%) were produced in the United States of America, three in Brazil (30%), two in India (20%), and one in Australia (10%).

Among the designs, there was a predominance of case studies (five – 50%), descriptive studies (four – 40%) and a qualitative study using Grounded Theory (one – 10%), composing the sample of studies analyzed.

In case studies, the occurrence of HELLP syndrome was found in second-time pregnant women (60%)^(18,19,20)^ and in first-time pregnant women (40%)^(21,22)^, aged between 24 and 28 years, who presented symptoms between 32 and 38 gestational weeks, as in all cases an emergency cesarean section was performed and all outcomes were live newborns who were discharged accompanied by their mothers. Only one study describes newborns with Apgar 6 and 7^(21)^; another study describes newborn results as Apgar 9 and 9^(18)^; and the others only mention healthy births^(19,20,22)^.

Among the descriptive studies, two studies stood out (40%) that addressed possible nursing diagnoses and interventions according to NANDA-NIC taxonomy^(23,24)^. A study (20%) described nursing care as a multidisciplinary team member^(25)^; a study described a care plan for care during the most critical moments of HELLP^(26)^; and a study described a physical and emotional care plan aimed at women and their families based on Grounded Theory^(27)^. Chart 2 presents the characteristics of the studies included in the review.

**Chart 2:** Characteristics of studies included in the review (n = 10) – Uberaba, MG, Brazil, 2023.

ID*	Author (s)	Year of publication	Country	Objectives	Population and sample size	Methodology	Results	Main findings
A1^(26)^	Adorno M, Maher-Griffiths C, Abadie HRG	2022	USA	Describe care in cases of HELLP syndrome.	NA	Descriptive study	Proposal for 11 specific nursing care measures to assist women with HELLP syndrome.	The study proposes a care plan for women with HELLP syndrome.
A2^(23)^	Beltrão HB, Brito CGR, Sousa DC, da Silva MEF, Brandão PF &dos Santos WA	2022	Brazil	Identify the main nursing diagnoses and nursing interventions for women with HELLP syndrome.	NA	Descriptive study based on a literature review and based on NANDA/NIC taxonomy	Eleven nursing diagnoses were described, eight of which were risk diagnoses and three were real. The diagnoses generated 55 possible nursing interventions.	The interventions are aimed at critical and intensive care for women, but also include care for the fetus/neonate and covering the emotional aspects of women and their families.
A3^(21)^	Bhatia R, Pathak V, Mor S & Gupta S	2020	India	Describe a case of a first-time pregnant woman diagnosed with eclampsia and HELLP syndrome.	01	Case study description	First-time pregnant woman, 26 years old, 38 weeks and four days of gestation, with a history of seizures for 48 hours. Upon admission, she was oliguric (100 ml diuresis/12 hours), with blood pressure of 200 x 110, fetal heart rate of 70 bpm and heart rate of 124 bpm. It abruptly progressed to cardiorespiratory arrest. She was immediately intubated and underwent cardiopulmonary resuscitation. Due to the persistence of cardiorespiratory arrest, an emergency cesarean section was performed. The newborn was born weighing 2,500 grams, asphyxiated, with Apgar scores 6/7. She received neonatal intensive care, including respiratory support with continuous positive airway pressure, monitoring of blood parameters, and administration of antibiotics to prevent infection. After the cesarean section, the patient was transferred to the Intensive Care Unit and received ventilatory support. Treatment included adequate antibiotic coverage, control of hypertension and monitoring of laboratory parameters such as platelet count and renal function. After 24 hours, the woman in labor was weaned from the ventilator. Dyas was discharged, without neurological sequel and in good condition.	The study points to the need for a multidisciplinary care team that is qualified to work in cardiopulmonary resuscitation. Timely cesarean section is also highlighted, in more serious cases, to guarantee better outcomes.
A4^(20)^	Cervantes R, Favre M& Carson-Romero C	2019	USA	Describe a case of second-time pregnant woman with epigastric pain.	01	Case study description	Second-time pregnant woman, with a previous miscarriage at screening, had intense epigastric pain and uterine contractions for three hours. Her cervix was closed and the fetus was tachycardic (fetal heart rate at 170 bpm). Intravenous therapy was started and a laboratory panel for pre-eclampsia was collected. Laboratory tests were highly suggestive of HELLP. An ultrasound was performed, which showed a large subcapsular hematoma in the liver. After an emergency cesarean section was indicated, a viable newborn was born without complications. During surgery, liver trauma was identified, which required suturing, which progressed to shock and disseminated intravascular coagulation. She required multiple transfusions, frequent visits to the surgical suite for reoperation and intubation for five days. She was discharged 12 days after giving birth.	Every complaint of epigastric pain must be carefully assessed and investigated, as it indicates the severity of the disease. It should be noted that pain precedes the laboratory test that indicates HELLP by several hours; therefore, in the complaint, a differential diagnosis must be carried out.
A5^(25)^	De Oliveira, RS, De Matos, IC, Da Silva, TBP, De Azevedo, NM, Andrade, M, &Do Espirito Santo, FH	2012	Brazil	Deepen understanding of HELLP syndrome and its evolution.	NA	Descriptive study based on narrative review	Eight studies made up the sample, five case studies, two reviews and one clinical trial. Predominantly laboratory diagnosis and the importance of investigating the syndrome in cases of hypertensive syndrome during pregnancy during prenatal care were observed. The importance of nursing consultation in screening and investigating symptoms is highlighted.	The importance of nursing consultation and in-depth investigation of symptoms is reinforced, especially for women with hypertensive syndrome during pregnancy.
A6^(19)^	DeshmukhA, Tarale S, Tembhre V & Pathade A	2022	India	Describe a case of a second-time pregnant woman with HELLP syndrome and severe anemia.	01	Case study description	Second-time pregnant woman, 24 years old, 37 weeks of gestation, complained of edema of the lower limbs bilaterally for a month, chest and abdominal pain, especially on the upper right side, vaginal bleeding, changes in vision, intolerance to heat, especially at night, but with cold palm, insomnia, weight loss due to nausea and vomiting, excessive sweating, dizziness, tremor, increased appetite and headache. Upon vaginal examination, the cervix was closed. Laboratory tests showed low hemoglobin values, a low platelet count and serum bilirubin within normal limits. Ultrasonography revealed a fetus with an age compatible with 31 weeks and altered cardiac flow. A cesarean section was performed under spinal anesthesia, however, during the procedure, the woman in labor developed a seizure and was immediately treated with magnesium sulfate, intravenous fluids and other medications, including oxytocin, vitamin K, tramadol and antibiotics. The newborn was born and cried immediately, indicating good vitality. However, due to low birth weight, the baby was transferred to the Neonatal Intensive Care Unit and received additional care, including continuous positive airway pressure, esophageal feedings every two hours, and administration of antibiotics.	The importance of investigating symptoms and considering the possibility of HELLP syndrome is highlighted, in addition to childbirth for better outcomes and care by a qualified multidisciplinary team.
A7^(18)^	Geake J, Dabscheck E &Reid D	2012	Australia	Describe a case of a second-time pregnant woman with cystic fibrosis who presented HELLP syndrome in the third trimester of pregnancy.	01	Case study description	Second-time pregnant woman, 26 years old, had cystic fibrosis as a comorbidity. Two weeks earlier, she experienced nausea and worsening respiratory function. After a week, she continued to experience nausea and worsening respiratory function. She opted for hospitalization, with blood pressure 153 x 89 mmHg, platelets at 101 thousand, alanine aminotransferase at 179 (3 x higher) and lactate dehydrogenase at 279 (<240). An ultrasound was performed with a suspected diagnosis of pre-eclampsia or HELLP. Two doses of corticosteroid therapy were applied within 12 hours, administering antihypertensive drugs. There was stability of the parameters, however, on the fifth day, she developed thrombocytopenia at 62 thousand, alanine aminotransferase at 118 and hemoglobin at 8.8 g/dl, with schistocytes in the blood. A diagnosis of HELLP syndrome was made. The cesarean section was performed ten hours after the result, and did not use magnesium sulfate. The newborn was born healthy, with Apgar scores 9/9. All parameters normalized postpartum.	The need for multidisciplinary teamwork, rigorous observation of symptoms and suspicion of HELLP syndrome, the importance of laboratory diagnosis, and urgent childbirth as definitive treatment were highlighted.
A8^(27)^	Kidner MC, Flanders-Stepans MB	2004	USA	Describe theory about the maternal experience of HELLP.	09	Qualitative study based on Grounded Theory, with nine HELLP survivors who had platelet counts below 100 thousand	HELLP‘S traumatic experiences are similar to those of a whirlwind. Even after recovering, the experience continues to be part of women’s emotional and physical aspects, described as common emotions: fear of death, frustration, anger and guilt, a universal feeling of loss of control and lack of information. In the speeches, the following categories emerged: Premonition; Symptoms; Feeling betrayed; The whirlwind; The losses; and No information and no control.	The study points to the need for empathetic care, involving everything from physical care to emotional care for women and their families.
A9^(24)^	Moraes MTS, Sousa RFO, Marcolino KMT, Davim RMB, CarvalhoCFS, Galvão CMB& Oliveira SX	2011	Brazil	Develop a proposal for a care plan for women with HELLP syndrome, taking into account the Nursing Process stages based on the main nursing diagnoses according to NANDA taxonomy.	NA	Descriptive study based on the search for diagnoses in NANDA taxonomy	Six risk diagnoses and four real diagnoses and 46 nursing interventions aimed at women with HELLP syndrome were identified.	The importance of systematizing nursing care in care planning as a guide for care for the team, women and family members is highlighted.
A10^(22)^	White A	2006	USA	Describe a case of a first-time pregnant woman diagnosed with HELLP syndrome.	01	Case study description	First-time pregnant woman, 28 years old, 32 weeks of gestation, who complained of extreme weakness. She requested emergency care due to severe pain in the upper quadrant of the abdomen and shoulders and edema of the lower limbs. When emergency services arrived, she was lying supine, pale, cold, and sweating profusely. She had blood pressure at 120x85 mmHg, heart rate at 140 bpm and respiratory rate at 28 bpm. Oxygen therapy 4l/minute and serum therapy were installed during transport. Upon admission, proteinuria was identified, indicating an emergency cesarean section. During the cesarean section, the obstetrician observed active hepatic hemorrhage. A ruptured hepatic hematoma and laceration involving the right lobe of the liver were found. Based on these findings, the diagnosis was expanded to HELLP syndrome. She received multiple transfusions and remained hospitalized in the CCU and on mechanical ventilation. She was discharged after 20 days of hospitalization. The newborn, who was born healthy, was discharged after ten days of life.	The study points to the need for physical care and emotional support for women and their families. Due to the severity of illnesses and possible losses, psychological aspects must be valued.

* ID – identification, article A followed by sequential number.

This review also allowed the creation of a chart (Chart 3) listing nursing care for women diagnosed with HELLP syndrome. Care is divided into five domains: prenatal; in the presence of symptoms/diagnosis; in the immediate/mediate postpartum period; family care; care at or during hospital discharge. In the prenatal domain, five nursing care measures are described; in the presence of symptoms/diagnosis, 18 precautions are described; in the immediate/mediate postpartum period, seven interventions are described; in caring for family members, six are described; and upon discharge or during its validity, three items are described. Thus, the present review identified 39 nursing care measures aimed at assisting women with HELLP syndrome.

**Chart 3:** Summary of nursing care for women with HELLP syndrome – Uberaba, MG, Brazil, 2023.

Nursing care for women with HELLP syndrome
**Prenatal care**
1. Carefully assess signs and symptoms of HELLP syndrome in women with hypertensive syndromes during prenatal care^(25)^ 2. Pay attention to the symptoms of HELLP syndrome and the need for investigation based on nursing consultation during prenatal care^(25)^ 3. Carefully assess and investigate complaints of epigastric and/or right hypochondrium pain in all pregnant women with complaints^(20)^ 4. Differentiate signs and symptoms of HELLP syndrome from common signs of pregnancy^(27)^ 5. Value clinical history and physical examination as important diagnostic tools^(27)^
**In the presence of symptoms/diagnosis**
1. Promote emergency transportation/care^(22)^ 2. Provide maternal hospitalization, preferably in a CCU^(23,26)^ 3. During maternal hospitalization, request a place in the neonatal CCU^(26)^ 4. Continuously monitor mother and fetus^(23,24,26)^, paying attention to vital signs. 5. Strictly control blood pressure, paying attention to systolic values above 160 mmHg and diastolic values above 110 mmHg^(26)^ 6. Puncture and maintain patent large-caliber venous access^(23,24)^ 7. Perform blood gas analysis for acid-base control according to medical prescription^(23,24)^ 8. Perform a rigorous water balance^(23,24)^ 9. Administer oxygen therapy^(23,24)^ 10. Provide ventilatory care, if necessary^(23,24)^ 11. Supervise mechanical ventilation, if necessary^(23,24)^ 12. Maintain strict monitoring of the level of consciousness by applying the Glasgow Coma Scale^(23,24)^ 13. Prevent seizures with the use of magnesium sulfate, if necessary^(26)^, as well as aspiration and possible injuries in case of seizures^(24)^ 14. Perform a detailed physical examination, including palpation of the right hypochondrium, inspection of the skin for signs of jaundice and/or pallor, observation of skin hydration, and presence of edema^(23,24)^ 15. If an emergency or immediate delivery is necessary, depending on gestational age, assess with the team the need and possibility of performing corticosteroid therapy^(26)^ 16. Plan the cesarean section in a timely manner^(18,19,21,26)^ 17. Use communication skills during care: reassure women; welcome with empathetic and work on active listening; always maintain eye contact during communication; and, if they are aware, inform them about the situation and allow them to make decisions^(23,27)^ 18. Promote care with a trained multidisciplinary team^(21)^, including training in cases of cardiorespiratory arrest^(18,21)^
**Immediate and immediate postpartum period**
1. Strictly assess/monitor lochiation every 15 minutes^(26)^ due to increased risk of bleeding 2. After maternal stabilization, promote bonding with newborn^(26)^ 3. As soon as the postpartum woman is stable, encourage breastfeeding or perform mechanical milking, if necessary^(26)^ 4. Observe the surgical incision for phlogistic signs and bleeding^(24)^ 5. Promote aseptic care when handling probes, drains and catheters^(24)^ 6. Promote skin hydration care and change position periodically, if necessary^(24)^ 7. Assess maternal conditions and advise on newborn care^(24)^
**Family care**
1. Assure the family that the woman and fetus are receiving the best care possible^(23)^ 2. Respect the privacy of each family member^(23)^ 3. Actively listen to family members’ concerns, questions and feelings^(23)^ 4. Provide information about the nursing care provided^(23)^ 5. Promote family involvement in decisions and care^(23)^ 6. Offer necessary support/support^(23)^
**Care at hospital discharge or during hospital stay**
1. Promote continuous emotional support in the postpartum period^(27)^ 2. Promote emotional/psychological care for postpartum women and their families in cases of fetal/neonatal loss^(22,27)^ 3. Refer at discharge to a support group to share and talk about their experience and be able to give it a new meaning^(27)^

## DISCUSSION

In the present review, diagnoses of HELLP syndrome were more frequent in women in the second decade of life, similar to other case studies and population studies^(28,29,30,31)^

Similarly, a study carried out in Alagoas showed a predominance of multiparous women in the cases identified^(30)^. A study in India showed a higher frequency in primiparous women^(29)^.

Regarding the gestational age at diagnosis, cases are most commonly observed in the third trimester of pregnancy, followed by few cases in the second trimester^(32)^, similarly to the reviewed studies and other studies^(28,29,31,33)^. The importance of prenatal nursing care is highlighted, in order to identify signs and intervene early, as a study carried out in Alagoas showed that in all cases women underwent prenatal consultations, with 66% having more than six consultations, as recommended by the Ministry of Health, and 44% had consultations, however fewer than recommended^(30)^.

A study pointed out the association between HELLP syndrome and antiphospholipid antibody syndrome (APS) and in these cases, an increase in premature births and fetal deaths. It is therefore recommended that screening and treatment be carried out if one condition or another is suspected. Treatment for APS improves neonatal prognosis by preventing or improving placental insufficiency^(32)^.

It is worth highlighting the importance of screening symptoms, starting from nursing consultation, whether during prenatal care or upon admission of the woman in labor. Sudden, abrupt and severe abdominal pain in the right upper quadrant was a symptom described by 30%^(34)^ to 78% of women^(35)^. Increased blood pressure was present in between 65%^(35)^ and 85%^(34)^ of women. Visual changes were described by 20%^(34)^ to 25% of women^(29)^.

In all studies reviewed, an emergency cesarean section was performed, similar to the outcomes presented in other studies, whose frequency ranged from 88%^(30)^ to 91.1%^(31)^. Only one study carried out in India showed that 84% of cases progressed to vaginal delivery^(29)^. It is noteworthy that completing the birth immediately and as quickly as possible contributes to a better maternal and neonatal prognosis, being indicated as a treatment for cases, which contributes to the increase in emergency cesarean sections in this case^(36)^.

A timely delay in delivery is indicated if gestational age is between 24 and 34 weeks and there is no maternal or fetal compromise, in order to perform corticosteroid therapy to allow fetal lung maturation^(36)^.

Among the studies reviewed, there were no cases of neonatal deaths, however, it is noted that it is a common outcome in severe conditions^(28,29,30,31)^. A meta-analysis showed that HELLP syndrome increases the risk of stillbirth by 1.56 times^(37)^.

Among live births, there is an increase in cases of prematurity^(29–31)^, with rates between 46%^(29)^ and 67%^(31)^ of births, increase in Apgar scores below seven^(29,30)^ and increase in cases of intrauterine growth restriction and low birth weight^(29–31)^. In this regard, nursing care is necessary to avoid prematurity and, when this is not possible, offer qualified nursing care and provide for the hospitalization of these newborns in specialized units in order to obtain better outcomes.

It is also noteworthy that a meta-analysis showed that HELLP syndrome contributed to an increase in the risk of acute renal failure by 4.87 times^(37)^ and by 3.7 times the risk of maternal death^(37)^. Among the case outcomes, hospitalization in Intensive Care Units^(28,33)^ for monitoring, polytransfusions^(28,29,33)^ and need for liver transplantation were observed^(33)^. Due to the critical nature, there is a need for rigorous monitoring and intensive care after birth^(32)^ to ensure a better maternal outcome.

The need to extend care beyond the treatment of the syndrome is highlighted to preserve women’s mental health, which can be undermined due to severity/criticality and possible losses. A review study pointed out mental health changes in women who had HELLP syndrome, with emphasis on an increased prevalence of depression, anxiety and post-traumatic stress syndrome. However, due to studies not controlling confounding factors, the results were inconclusive^(38)^. Therefore, care must be taken regarding the preservation or restoration of these women’s mental health, as mentioned in the reviewed articles.

As previously described, HELLP syndrome is a serious condition that, if left untreated, can lead to maternal death. Therefore, when thinking about care, it is necessary to think about possible delays that could prevent women from receiving adequate and necessary care. Based on the three delays model, it is necessary to rethink care in the three components. The first delay occurs in the decision to seek care. This first pillar is influenced by the refusal of women and family members^(39)^. Thus, strengthening educational practices and qualified prenatal care can be fundamental to avoid it.

The second delay refers to women’s route and access to health services, which range from geographic factors to transport and infrastructure. The third delay consists of receiving adequate care when there is access to health services^(39)^. At these points, it is necessary to guarantee access to networked healthcare, with its different levels of care. The importance of health services thinking about the phenomenon of delays to analyze solutions for maternal morbidity and mortality is reinforced.

Furthermore, it is necessary to consider the critical nodes of care. The study proposes a care plan based on evidence, however we identified the relevance of: strengthening care at the entrance to maternity wards, reinforcing the reception methodology and risk classification as a qualification strategy; ensuring adequate staffing so that the presence of nurses caring for a woman with HELLP does not make it impossible for nurses to care for other women in labor; and strengthening continuing education actions with a view to qualified interprofessional shared care.

The importance of taxonomies for nurses’ decision-making is highlighted^(40)^. Although only two studies in the present review described diagnoses and interventions and there are no specific diagnoses or interventions for the condition, taxonomies contribute to care planning^(24)^ as well as effective decision-making in the face of an obstetric emergency^(22)^, as is the case with HELLP syndrome.

However, there is a lack of studies that address nursing care in cases of HELLP syndrome, which compromises the discussion of the findings and is a limitation of this study. Furthermore, there are few studies published on the topic today, which compromises the comparability of results. At the same time, the implications of this review for practice are highlighted, as care inherent to each phase of the pregnancy-puerperal cycle in which the syndrome is detected is analyzed and presented.

## CONCLUSION

The present review highlighted the magnitude of the syndrome; however, studies are scarce. There was a predominance of case studies, followed by descriptive studies. The review made it possible to identify 39 nursing care measures aimed at assisting women with HELLP syndrome.
