# Supplementary material for: Nursing care for women with HELLP syndrome: a scoping review
Source: Rev Esc Enferm USP. 2024 Aug 12;58:e20240116. doi: 10.1590/1980-220X-REEUSP-2024-0116en (PMC11321456; doi:10.1590/1980-220X-REEUSP-2024-0116en)
Supplement: Translation

# Translation: Cuidados de enfermagem a mulheres com síndrome HELLP: scoping review

**DOI:** 10.1590/1980-220X-REEUSP-2024-0116pt

**Authors:** Pâmela Silva Arduini, Cynthya Viana de Resende, Jéssica Aparecida da Silva, Mariana Torreglosa Ruiz

**Affiliations:** 1 Universidade Federal do Triângulo Mineiro, Uberaba, MG, Brasil.; 1 Universidade Federal do Triângulo Mineiro, Programa de Pós-Graduação em Atenção à Saúde, Uberaba, MG, Brasil.

**Keywords:** Síndrome HELLP, Cuidado de Enfermagem, Enfermagem Obstétrica

## Abstract

**Objetivo:**

Mapear evidências sobre assistência de enfermagem às mulheres com síndrome HELLP.

**Método:**

Scoping review, com buscas realizadas em maio de 2023, independentemente, nas bases PubMed/MEDLINE, LILACS, Scopus, EMBASE, Web of Science, CINAHL, Catálogo de Teses e Dissertações da CAPES e Cochrane Library, correlacionando os descritores HELLP Syndrome, Nursing Care e Obstetric Nursing e seus sinônimos, sem delimitação de tempo e idioma. A seleção foi realizada por três pesquisadores independentemente e resolvida por consenso.

**Resultados:**

Das 129 publicações, foram selecionadas dez, que compuseram a amostra final. As publicações datam de 2004 a 2022, com predomínio do idioma inglês e de estudos de casos clínicos. Observou-se maior ocorrência da síndrome em secundigestas na segunda década de vida, com idade gestacional a partir de 32 semanas, que tiveram como desfecho cesárea de emergência, e todos os recém-nascidos receberam alta acompanhados das mães. Resgataram-se estudos que descreviam diagnósticos de enfermagem e que focavam em cuidados assistenciais de enfermagem. A partir da revisão, identificaram-se 39 cuidados de enfermagem.

**Conclusão:**

A presente revisão apontou a magnitude da síndrome, contudo possui escassez de estudos.

## INTRODUÇÃO

De acordo com a Organização Mundial da Saúde (OMS), as síndromes hipertensivas na gestação são responsáveis por um décimo das mortes maternas^(1)^. No cenário nacional, dados do Departamento de Informática do Sistema Único de Saúde (DATASUS) apontam que, no período compreendido entre 1996 e 2021, registraram-se mais de 45 mil óbitos maternos e 2.808 óbitos tardios, totalizando 48.301 óbitos maternos. Desses, 62% foram decorrentes de causas obstétricas diretas, e as síndromes hipertensivas representaram 34,4% de todas as causas (16.622 óbitos), sendo a eclâmpsia o primeiro motivo de morte materna no país^(2)^.

Revisão de estudos brasileiros sobre pré-eclâmpsia e eclâmpsia apontou frequência de pré-eclâmpsia de 6,7% e, de eclâmpsia, entre 1,7% e 6,2%, e a síndrome HELLP representou a evolução de um em cada 30 casos de pré-eclâmpsia, sendo uma condição pouco relatada, embora represente maior gravidade da evolução dos quadros^(3)^.

A pré-eclâmpsia se caracteriza por aumento pressórico após a vigésima semana gestacional, associado à proteinúria, que pode ser resolvido após o nascimento ou evoluir com complicações e sequelas. Já a eclâmpsia consiste em quadro que cursa com convulsões não atribuídas a causas neurológicas em pacientes com diagnóstico prévio de pré-eclâmpsia, sendo classificada como uma complicação da pré-eclâmpsia. A síndrome HELLP é um agravamento dos quadros de pré-eclâmpsia cursando com hemólise (H), elevação da das enzimas hepáticas (EL) e plaquetopenia grave (LP), sendo considerada a evolução de maior gravidade das síndromes hipertensivas gestacionais^(4,5)^.

Considera-se aumento pressórico na gestação valores de pressão arterial sistólica (PAS) maior ou igual a 140 mmHg e/ou de diastólica (PAD) maior ou igual a 90 mmHg, aferidos com técnica adequada e confirmados por duas medidas com intervalo de quatro horas. Obtido valor de PAS superior a 160 mmHg e/ou de PAD superior a 110 mmHg, recomenda-se nova aferição em 15 minutos e, se mantidos os valores, iniciar tratamento imediatamente. Já a proteinúria é confirmada laboratorialmente pela presença de 300 mg ou mais de proteínas em urina de 24 horas ou resultado da relação proteína/creatinina urinária igual ou superior a 0,3, ou, caso não seja possível a realização do exame de 24 horas, a presença de pelo menos uma cruz em amostra de urina isolada^(4,5)^.

A primeira descrição sobre a síndrome HELLP data de 1982, pelo médico Louis Weinstein^(6)^, que observou o surgimento da sintomatologia a partir de 20 semanas gestacionais assim como suas primeiras manifestações no puerpério. A síndrome tem como sintomatologia frequente o mal-estar súbito, ocorrência de náuseas e vômitos, e dor abdominal abrupta e intensa no quadrante superior direito do abdome e/ou epigástrica, sendo a dor o sintoma característico da síndrome. Geralmente, o quadro associa-se à complicação da pré-eclâmpsia^(7)^, contudo, em 15% a 20% dos casos, podem ocorrer sem aumento hipertensivo ou sem proteinúria^(8)^.

Para conclusão do diagnóstico, são necessários exames laboratoriais, constituindo-se em critérios: 1. Hemólise (esfregaço anormal sugestivo de anemia hemolítica microangiopática caracterizada por presença de esquistócitos); 2. Bilirrubina total acima de 1,2 mg/dl; 3. Lactato desidrogenase acima de 6.000 U/l ou hepatoglobina menor do que o limite inferior para normalidade; 4. Enzima hepática alanina aminotransferase com valor duas vezes superior ao limite da normalidade; e 5. Contagem de plaquetas inferior a 100.000/μl. Desses critérios, a presença de um a dois sugere possibilidade de HELLP, e três critérios definem o diagnóstico^(9)^.

Entre as possíveis complicações da síndrome, são frequentes edema pulmonar, lesão renal reversível ou não, necessidade de politransfusões, internação em Centro de Terapia Intensivo (CTI) e coagulação intravascular disseminada^(8,9)^, o que confere maior gravidade aos casos.

A síndrome HELLP caracteriza-se como um quadro grave e agudo, consistindo em uma emergência obstétrica. Acomete uma mulher a cada 45.000 nascimentos vivos, representando de 0,1% a 0,9% das complicações durante o ciclo gravídico-puerperal, sendo que de 10% a 30% dos quadros se apresenta como uma complicação da pré-eclâmpsia^(7)^. Cerca de 45% dos casos de HELLP evoluem com gravidade^(10)^. Todos os casos requerem cuidados intensivos, e 1% evolui para ruptura hepática^(11)^, condição que constitui risco de mortalidade, principalmente quando as plaquetas atingem níveis inferiores a 50.000^(10,11)^.

Na maioria dos casos, a etiopatogenia é desconhecida. No entanto, a nuli ou multiparidade, idade superior a 30 anos, história de síndrome HELLP em gestação prévia, hipertensão arterial crônica, diabetes com diagnóstico pré-gestacional, cardiopatias, obesidade, doenças hepáticas crônicas, alterações placentárias e anormalidades congênitas são descritos como fatores de risco para a síndrome HELLP^(12)^.

Além do impacto negativo e possíveis complicações na vida da mulher^(13,14)^, as síndromes hipertensivas no período gravídico-puerperal estão associadas com a prematuridade, e essa é a principal causa de morte em crianças menores de cinco anos de idade^(15)^.

A assistência pré-natal de qualidade é um fator protetor para as complicações da síndrome, que embora não possa ser prevenida, quanto mais precoce a realização do diagnóstico, maior a possibilidade de redução de danos. A interrupção da gestação está indicada como tratamento assim que estabilizado o quadro, ou seja, não deve ser intempestiva, nem imediata, mas planejada para evitar desfechos negativos^(5)^. Dessa forma, devido à sua complexidade e ao alto potencial de complicações, identifica-se a importância de assistência de enfermagem qualificada e pautada em evidências científicas.

A presente pesquisa visa apresentar a síntese do conhecimento sobre os cuidados assistenciais de enfermagem às mulheres com síndrome HELLP, de forma que permita ao enfermeiro a tomada de decisão segura e precisa baseada em resultados prévios descritos na literatura. Diante do exposto, este estudo teve como objetivo mapear evidências sobre a assistência de enfermagem às mulheres com síndrome HELLP.

## MÉTODO

### Tipo de Estudo

*Scoping review*, desenvolvida com base nas recomendações do JBI^(16)^. Desse modo, percorreram-se as etapas de: (1) estabelecimento do título e da pergunta de revisão a partir do mnemônico do PCC, sendo que P: População, C: Conceito e C: Contexto; (2) exploração do estado da arte do problema de investigação com redação da introdução da revisão; (3) definição dos critérios de inclusão; (4) delineamento da estratégia de busca (fontes, descritores e referências manuais a partir da leitura das publicações selecionadas); (5) seleção da fonte de evidência (examinador e protocolo); (6) seleção dos artigos – processo guiado pelo fluxograma *Preferred Reporting Items for Systematic Reviews and Meta-Analysis* (PRISMA-ScR)^(17)^; (7) extração dos dados; (8) análise das evidências; e (9) apresentação dos resultados de forma tabular e por meio de mapeamento descritivo. Protocolo foi registrado no *Open Science Framework* (https://osf.io/d8kup).

### Estratégia de Busca

Para elaborar a questão de revisão, utilizou-se o mnemônico PCC, sendo que a População (P) incluiu mulheres gestantes ou puérperas, Conceito (C) incluiu cuidados de enfermagem, e Contexto (C) incluiu síndrome HELLP. Dessa forma, a questão de revisão foi: quais as evidências sobre cuidados de enfermagem a mulheres com síndrome HELLP?

As buscas foram realizadas em maio de 2023, de forma independente, por dois revisores, um mestrando e um doutor. Um revisor tem experiência com estratégia de busca e curso de treinamento para *scoping reviews*, e ambos são especialistas na área da saúde materno-infantil. A busca foi validada por um bibliotecário. Utilizaram-se os descritores *HELLP Syndrome, Nursing Care e Obstetric Nursing*. As bases de dados *US National Library of Medicine National Institutes of Health* (MEDLINE/PubMed), *Web of Science* (WoS), *Excerpta Medica DataBASE* (EMBASE), *SciVerse Scopus*, Literatura Latino-Americana e do Caribe em Ciências da Saúde (LILACS), Catálogo de Teses e Dissertações da CAPES e *Cochrane Library* foram selecionadas para consulta, correlacionando os descritores *HELLP Syndrome, Nursing Care* e *Obstetric Nursing*. Não foram aplicados filtros de data, idioma e/ou desenho de estudo. As estratégias de busca encontram-se descritas no Quadro 1, com o retorno numérico obtido.

**Quadro 1:** Estratégias de buscas nas bases de dados e retorno numérico obtido – Uberaba, MG, Brasil, 2023.

Base de dados	Estratégia de busca	n
**PubMed**	(((“HELLP Syndrome” [Mesh] OR Syndrome, HELLP OR Hemolysis, Elevated Liver Enzymes, Lowered Platelets) AND (“Nursing Care” [Mesh] OR Care, Nursing OR Management, Nursing Care OR Nursing Care Management OR “Obstetric Nursing” [Mesh] OR Nursing, Obstetric OR Nursings, Obstetric OR Obstetric Nursings OR Nursing, Obstetrical OR Nursings, Obstetrical OR Obstetrical Nursings OR Obstetrical Nursing)	**25**
**EMBASE**	(‘HELLP Syndrome’/exp OR ‘HELLP (hemolysis, elevated liver enzymes and low platelets) syndrome’ OR ‘hemolysis, elevated liver enzymes, and low platelet count syndrome’ OR ‘hemolysis, elevated liver enzymes, and low platelets syndrome’ OR ‘hemolysis, elevated liver enzymes, low platelet count syndrome’ OR ‘syndrome HELLP’ OR ‘HELLP syndrome’) AND (‘Obstetrical nursing’/exp OR ‘obstetric nursing’ OR ‘obstetrics nursing’ OR ‘obstetrical nursing’ OR ‘Nursing’/exp OR ‘hospital nursing service’ OR ‘nursing service’ OR ‘nursing service, hospital’ OR ‘nursing services’ OR ‘nursing support’ OR ‘nursing, private duty’ OR ‘nursing, supervisory’ OR ‘office nursing’ OR ‘private duty nursing’ OR ‘supervisory nursing’ OR ‘nursing’) AND [embase]/lim NOT ([embase]/lim AND [medline]/lim)	**28**
**Scopus**	ALL (“HELLP Syndrome”) AND TITLE-ABS-KEY (*nursing AND care* OR *obstetric AND nursing* )	**54**
**WoS**	(ALL=(HELLP syndrome*)) AND ALL=(Nursing care*) AND (ALL=(HELLP syndrome*)) AND ALL=(obstetric nursing*)	**27**
**LILACS**	(tw:(“Síndrome HELLP” OR “HELLP Syndrome” OR “HELLP syndrome” OR C12.050.703.395.186 )) AND (tw:(“Cuidados de Enfermagem” OR “Nursing Care” OR “Atención de Enfermería” OR “Soins infirmiers” OR “Assistência de Enfermagem” OR “Atendimento de Enfermagem” OR “Cuidado de Enfermagem” OR “Gestão da Assistência de Enfermagem” OR “Sistematização da Assistência de Enfermagem” OR “E02.760.611” “OR N02.421.533” OR “Enfermagem Obstétrica” OR “Obstetric Nursing” OR “Enfermería Obstétrica” OR “Soins infirmiers em obstétrique” OR “H02.478.676.570” OR “N02.421.533.571”)) AND (db:(LILACS))	–
**CAPES**	Síndrome HELLP	**88**
**Cochrane**	HELLP syndrome	**07**

Para ampliar o alcance das buscas, foram adicionadas variações terminológicas nos diferentes idiomas, sinônimos dos descritores controlados e os operadores booleanos *AND*, para ocorrência simultânea de assuntos, e operadores booleanos *OR*, para ocorrência de um outro assunto, conforme sintetizado no Quadro 1.

Foram incluídos estudos que descrevessem cuidados de enfermagem a mulheres com síndrome HELLP, sem delimitação de tempo ou idioma. Foram excluídos artigos duplicados nas bases, artigos de opinião, editoriais, consenso(s), carta resposta ou cartas ao editor, resumos apresentados em anais de eventos e aqueles que não respondessem à questão de revisão. Ressalta-se que o nível de evidência não foi considerado critério de exclusão, por se tratar de tema pouco explorado na literatura. Adotou-se a metodologia PRISMA^(17)^ para seleção dos artigos, e a mesma foi ilustrada em fluxograma (Figura 1).

**Figura 1:** Fluxograma PRISMA de seleção dos estudos. Uberaba, MG, Brasil, 2023. Fonte: elaborado pelos autores com base no PRISMA SCr 2020^(17)^.

A seleção dos estudos foi realizada, de modo independente, por três pesquisadores, e as discordâncias foram resolvidas por consenso, sem necessidade de acréscimo de novo revisor nessa etapa. A análise inicial dos estudos selecionados foi realizada a partira da leitura do título e resumo, seguida de leitura exaustiva na íntegra para a seleção final dos estudos. A Figura 1 ilustra o processo de seleção dos estudos.

Na busca, foram localizadas 129 publicações. Na primeira etapa, as duplicatas foram removidas (n = 40) e 70 artigos foram excluídos após a leitura dos títulos e resumos, por não retratarem o tema de estudo ou não possuírem desenho/tipo de estudo adequado para inclusão, conforme os critérios estabelecidos. Das 19 publicações analisadas na íntegra, dez foram selecionadas após a leitura das mesmas. Dessa forma, a amostra final foi composta por dez estudos.

A extração de dados foi realizada por três pesquisadores, independentemente. Foram extraídas informações padronizadas pelo JBI, como autoria, ano, país produtor, objetivos, população e tamanho amostral, metodologia utilizada, desfechos, principais resultados que respondem à questão de revisão e risco de viés. Os dados extraídos foram tabulados e apresentados através de síntese narrativa.

## RESULTADOS

Dez estudos compuseram a revisão, publicados entre 2004 e 2022 (30%), sete (70%) publicados em inglês e três (30%) em português. Quatro estudos (40%) foram produzidos nos Estados Unidos, três, no Brasil (30%), dois, na Índia (20%), e um, na Austrália (10%).

Entre os desenhos, verificou-se predominância de estudos de caso (cinco – 50%), estudos descritivos (quatro – 40%) e um estudo qualitativo com uso da Teoria Fundamentada nos Dados (um – 10%), compondo a amostra dos estudos analisados.

Nos estudos de caso, verificou-se a ocorrência da síndrome HELLP em secundigestas (60%)^(18–20)^ e em primigestas (40%)^(21,22)^, com idade entre 24 e 28 anos, que apresentaram sintomas entre 32 e 38 semanas gestacionais, sendo que, em todos os casos, realizou-se cesárea de emergência e todas tiveram como desfechos recém-nascidos vivos que receberam alta acompanhados das mães. Apenas um estudo descreve recém-nascido com escores de Apgar 6 e 7^(21)^; outro estudo descreve como resultado neonato com Apgar 9 e 9^(18)^; e os demais mencionam apenas nascimentos saudáveis^(19,20,22)^.

Entre os estudos descritivos, destacaram-se dois estudos (40%) que abordaram os possíveis diagnósticos e intervenções de enfermagem segundo a taxonomia NANDA-NIC^(23,24)^. Estudo (20%) descreveu os cuidados de enfermagem enquanto membro da equipe multidisciplinar^(25)^; estudo descreveu plano de cuidados para assistência durante os momentos mais críticos da HELLP^(26)^; e estudo descreveu um plano de cuidados físicos e emocionais voltados para a mulher e seus familiares a partir da Teoria Fundamentada nos Dados^(27)^. No Quadro 2, são apresentadas as características dos estudos inclusos na revisão.

**Quadro 2:** Características dos estudos inclusos na revisão (n = 10) – Uberaba, MG, Brasil, 2023.

ID*	Autor(es)	Ano de publicação	País	Objetivos	População e tamanho amostral	Metodologia	Resultados	Principais achados
A1^(26)^	Adorno M, Maher-Griffiths C, Abadie HRG	2022	Estados Unidos	Descrever cuidados em casos de síndrome HELLP.	NA	Estudo descritiv o	Proposta de 11 cuidados de enfermagem específicos para assistência a mulheres com síndrome HELLP.	O estudo propõe um plano de cuidados assistenciais à mulher com síndrome HELLP.
A2^(23)^	Beltrão HB, Brito CGR, Sousa DC, da Silva MEF, Brandão PF & dos Santos WA	2022	Brasil	Identificar os principais diagnósticos de enfermagem e intervenções de enfermagem para mulheres com síndrome HELLP.	NA	Estudo descritivo a partir de revisão na literatura e fundamentado na taxonomia NANDA/NIC.	Descreveram-se 11 diagnósticos de enfermagem, sendo oito diagnósticos de risco e três reais. Os diagnósticos geraram 55 possíveis intervenções de enfermagem.	As intervenções são voltadas para cuidados críticos e intensivos para a mulher, mas também compreendem cuidados com feto/neonato e abrangência dos aspectos emocionais da mulher e família.
A3^(21)^	Bhatia R, Pathak V, Mor S & Gupta S	2020	Índia	Descrevercaso de primigesta com diagnóstico de eclâmpsia e síndrome HELLP.	01	Descrição de estudo de caso	Primigesta, 26 anos, com 38 semanas e quatro dias de gestação, com história de convulsões há 48 horas. Na admissão, encontrava-se oligúrica (100 ml de diurese/ 12 horas), com pressão arterial de 200 x 110, batimentos cardíacos fetais de 70 bpm e frequência cardíaca de 124 bpm. Evoluiu abruptamente para parada cardiorrespiratória. Foi imediatamente intubada e submetida à reanimação cardiopulmonar. Devido à persistência de parada cardiorrespiratória, realizou-se cesárea de urgência. O neonato nasceu com 2.500 gramas, asfixiado, com escores de Apgar 6/7. Recebeu cuidados intensivos neonatais, incluindo suporte respiratório com pressão positiva contínua nas vias aéreas, monitoramento dos parâmetros sanguíneos e administração de antibióticos para prevenção de infecção. Após a cesariana, a paciente foi transferida para a Unidade de Terapia Intensiva e recebeu suporte ventilatório. O tratamento incluiu cobertura antibiótica adequada, controle da hipertensão e acompanhamento dos parâmetros laboratoriais, como contagem de plaquetas e função renal. Após 24 horas, a puérpera foi desmamada do ventilador. O binômio recebeu alta, sem sequelas neurológicas e em bom estado.	O estudo aponta para a necessidade de equipe multidisciplinarna assistência que possua capacitação para atuar na reanimação cardiopulmonar . Também destaca-se a cesárea em tempo hábil, em casos de maior gravidade, para garantia de melhores desfechos.
A4^(20)^	Cervantes R, Favre M & Carson-Romero C	2019	Estados Unidos	Descrever caso de secundigestas com dor epigástrica.	01	Descrição de estudo de caso	Secundigesta, com aborto anterior na triagem, apresentava dor epigástrica intensa e contrações uterinas há três horas. O colo estava fechado e o feto estava taquicárdico (batimentos cardíacos fetais em 170 bpm). Iniciou-se terapia endovenosa e foi colhido painel laboratorial para pré-eclâmpsia. Os exames laboratoriais eram altamente sugestivos para HELLP. Realizou-se ultrassonografia, que mostrou grande hematoma subcapsular no fígado. Após indicada cesárea de emergência, recém-nascido viável nasceu sem intercorrências. Durante a cirurgia, identificou-se trauma hepático, que necessitou de sutura, evoluiu com choque e coagulação intravascular disseminada. Necessitou de múltiplas transfusões, idas frequentes ao bloco cirúrgico para reabordagem e de intubação por cinco dias. Recebeu alta com 12 dias após o parto.	Toda queixa de dor epigástrica deve ser cuidadosamente avaliada e investigada, pois indica gravidade da doença. Ressalta-se que a dor precede o exame laboratorial que aponta HELLP em várias horas; portanto, na queixa, deve-se realizar o diagnóstico diferencial.
A5^(30)^	De Oliveira, RS, De Matos, IC, Da Silva, TBP, De Azevedo, NM, Andrade, M, & Do Espirito Santo, FH	2012	Brasil	Aprofundar a compreensão sobre a síndrome HELLP e sua evolução.	NA	Estudo descritivo a partir de revisão narrativa	Oito estudos compuseram a amostra, sendo cinco estudos de caso, duas revisões e um ensaio clínico. Observaram-se diagnóstico predominantemente laboratorial e a importância da investigação da síndrome nos casos de síndrome hipertensiva na gestação durante o pré-natal. Destaca-se a importância da consulta de enfermagem na triagem e investigação da sintomatologia.	Reforça-se a importância da consulta de enfermagem e da investigação dos sintomas em profundidade, em especial às mulheres com síndrome hipertensiva na gestação.
A6^(19)^	DeshmukhA, Tarale S, Tembhre V & Pathade A	2022	Índia	Descrevercaso de secundigesta com síndrome HELLP e anemia severa.	01	Descrição de estudo de caso	Secundigesta, 24 anos, 37 semanas de gestação, apresentou queixa de edema de membros inferiores bilateralmente há um mês, dor torácica e abdominal, especialmente no lado superior direito, sangramento vaginal, alterações na visão, intolerância ao calor, especialmente à noite, mas com palma da mão fria, insônia, perda de peso devido a náuseas e vômitos, suor excessivo, tontura, tremor, aumento do apetite e cefaleia. Ao toque vaginal, o colo do útero estava fechado. Exames laboratoriais mostraram valores de hemoglobina baixos, contagem de plaquetas baixa e bilirrubina sérica dentro dos limites normais. A ultrassonografia revelou um feto com idade compatível com 31 semanas e alteração de fluxo cardíaco. Realizou-se cesárea sob raquianestesia, no entanto, durante o procedimento, a parturiente apresentou convulsão e foi tratada imediatamente com sulfato de magnésio, fluidos intravenosos e outros medicamentos, incluindo ocitocina, vitamina K, tramadol e antibióticos. O neonato nasceu e chorou imediatamente, indicando boa vitalidade. No entanto, devido ao baixo peso ao nascer, o bebê foi transferido para a Unidade de Terapia Intensiva Neonatal e recebeu cuidados adicionais, incluindo pressão positiva contínua nas vias aéreas, alimentação esofágica a cada duas horas e administração de antibióticos.	Ressalta-se a importância da investigação de sintomas e de se aventar a possibilidade de síndrome HELLP, além do parto para melhores desfechos e do atendimento por equipe multidisciplinar capacitada.
A7^(18)^	Geake J, Dabscheck E & Reid D	2012	Austrália	Descrevercaso de secundigesta com fibrose cística que apresentousíndrome HELLP no terceiro trimestre gestacional.	01	Descrição de estudo de caso	Secundigesta, 26 anos, tinha como comorbidade a fibrose cística. Duas semanas antes, apresentou náuseas e piora da função respiratória. Com uma semana, manteve náuseas e piora da função respiratória. Optou-se por internação, com pressão arterial 153 x 89 mmHg, plaquetas a 101 mil, alanina aminotransferase a 179 (3 x superior) e lactato desidrogenase a 279 (< 240). Realizou-se ultrassonografia com suspeita diagnóstica de pré-eclâmpsia ou HELLP. Aplicaram-se duas doses de corticoterapia em 12 horas, administrando anti-hipertensivos. Houve estabilidade dos parâmetros, contudo, no quinto dia, evoluiu com plaquetopenia a 62 mil, alanina aminotransferase a 118 e hemoglobina a 8,8 g/dl, com esquistócitos no sangue. Foi fechado diagnóstico de síndrome HELLP. A cesárea foi realizada após dez horas do resultado, e não utilizou sulfato de magnésio. O recém-nascido nasceu saudável, com escores de Apgar 9/9. Todos os parâmetros normalizaram-se pós-parto.	Destacou-se a necessidade de: trabalho em equipe multidisciplinar; observar rigorosamente sintomas e suspeitar de síndrome HELLP; a importância do diagnóstico laboratorial; parto urgente como tratamento definitivo.
A8^(27)^	Kidner MC, Flanders-Stepans MB	2004	Estados Unidos	Descrever teoria sobre a experiência materna da HELLP.	09	Estudo qualitativo a partir da Teoria Fundamentada nos Dados, com nove sobreviventes à HELLP que tiveram contagem de plaquetas inferior a 100 mil	As experiências traumáticas da HELLP são semelhantes às de um redemoinho. Mesmo recuperadas, a experiência continua a fazer parte do emocional e físico das mulheres, descritas como emoções comuns o medo da morte, frustração, raiva e culpa, sentimento universal de perda de controle e de falta de informações. Nas falas, emergiram as categorias: Premonição; Sintomas; Sentir-se traída; O redemoinho; As perdas; e Sem informações e sem controle.	O estudo aponta para necessidade de cuidado empático, envolvendo desde cuidados físicos aos cuidados emocionais com a mulher e sua família.
A9^(24)^	Moraes MTS, Sousa RFO, Marcolino KMT, Davim RMB, CarvalhoCFS, Galvão CMB & Oliveira SX	2011	Brasil	Elaborar uma proposta de plano assistencial às mulheres com síndrome HELLP, tendo em vista as etapas do Processo de Enfermagem baseado nos principais diagnósticos de enfermagem segundo a taxonomia NANDA.	NA	Estudo descritivo a partir da busca de diagnósticos na taxonomia NANDA	Identificaram-se seis diagnósticos de risco e quatro diagnósticos reais e 46 intervenções de enfermagem voltados à mulher com síndrome HELLP.	Ressalta-se a importância da sistematização da assistência de enfermagem no planejamento da assistência como norteador do cuidado para equipe, mulheres e familiares.
A10^(22)^	White A	2006	Estados Unidos	Descrevercaso de primigesta com diagnóstico de síndrome HELLP.	01	Descrição de estudo de caso	Primigesta, 28 anos, 32 semanas de gestação, que se queixava de fraqueza extrema. Solicitou atendimento de emergência por dor intensa no quadrante superior do abdome e em ombros e edema de membros inferiores. Quando o serviço de emergência chegou, ela estava deitada em decúbito dorsal, pálida, fria, com sudorese intensa. Ela tinha pressão arterial a 120x85 mmHg, frequência cardíaca a 140 bpm e frequência respiratória a 28 ipm. Instalada oxigenoterapia 4l/minuto e soroterapia durante o transporte. Na internação, identificou-se proteinúria, indicando cesárea de emergência. Durante a realização da cesárea, o obstetra observou hemorragia hepática ativa. Constatou-se hematoma hepático roto e laceração envolvendo o lobo direito do fígado. Com base nessas descobertas, o diagnóstico foi expandido para síndrome HELLP. Foi politransfundida, permaneceu internada em CTI e em ventilação mecânica. Recebeu alta com 20 dias de internação. O neonato, que nasceu saudável, recebeu alta após dez dias de vida.	O estudo aponta para a necessidade de cuidados físicos e de apoio emocional para as mulheres e suas famílias. Devido à gravidade das doenças e possíveis perdas, os aspectos psicológicos devem ser valorizados.

* Id – identificação, A de artigo seguido de número sequencial.

A presente revisão permitiu ainda a confecção de um quadro (Quadro 3) listando os cuidados de enfermagem a mulheres com diagnóstico de síndrome HELLP. Os cuidados são divididos em cinco domínios: pré-natal; na vigência de sintomas/diagnóstico; no puerpério imediato/mediato; cuidados com familiares; cuidados na alta hospitalar ou na sua vigência. No domínio pré-natal, são descritos cinco cuidados de enfermagem; na vigência de sintomas/diagnóstico, são descritos 18 cuidados; no puerpério imediato/mediato, são descritas sete intervenções; no cuidado com familiares, são descritos seis; e na alta ou na sua vigência, são descritos três itens. Assim, a presente revisão permitiu identificar 39 cuidados de enfermagem voltados à assistência à mulher com síndrome HELLP.

**Quadro 3:** Sumário de cuidados de enfermagem a mulheres com síndrome HELLP – Uberaba, MG, Brasil 2023.

Cuidados de enfermagem a mulheres com síndrome HELLP
**Pré-natal**
1. Avaliar criteriosamente sinais e sintomas de síndrome HELLP em mulheres com síndromes hipertensivas durante o pré-natal^(25)^ 2. Atentar-se para sintomatologia da síndrome HELLP e a necessidade de investigação a partir da consulta de enfermagem durante a assistência pré-natal^(25)^ 3. Avaliar e investigar cuidadosamente a queixa de dor epigástrica e/ou no hipocôndrio direito em todas as gestantes com queixa^(20)^ 4. Diferenciar sinais e sintomas de síndrome HELLP de sinais comuns da gestação^(27)^ 5. Valorizar história clínica e exame físico como importantes ferramentas diagnósticas^(27)^
**Na vigência de sintomas/diagnóstico**
1. Promover transporte/assistência de emergência^(22)^ 2. Providenciar internação materna preferencialmente em CTI^(23,26)^ 3. Na vigência de internação materna, solicitar vaga de CTI neonatal^(26)^ 4. Monitorar continuamente mãe e feto^(23,24,26)^, atentando-se para os sinais vitais. 5. Controlar rigorosamente a pressão arterial, atentando para valores de sistólica superiores a 160 mmHg e de diastólica superiores a 110 mmHg^(26)^ 6. Puncionar e manter acesso venoso de grosso calibre pérvio^(23,24)^ 7. Realizar gasometria para o controle ácido-básico, de acordo com prescrição médica^(23,24)^ 8. Realizar balanço hídrico rigoroso^(23,24)^ 9. Administrar oxigenoterapia^(23,24)^ 10. Prover assistência ventilatória, se necessário^(23,24)^ 11. Supervisionar a ventilação mecânica, se necessário^(23,24)^ 12. Manter vigilância rigorosa do nível de consciência por meio da aplicação da escala de coma de Glasgow^(23,24)^ 13. Prevenir convulsões com uso de sulfato de magnésio, se necessário^(26)^, assim como aspiração e possíveis lesões em caso de convulsões^(24)^ 14. Realizar exame físico detalhado, incluindo palpação do hipocôndrio direito, inspeção da pele para sinais de icterícia e/ou palidez, observação da hidratação da pele e presença de edemas^(23,24)^ 15. Se necessidade de parto de emergência ou imediato, a depender da idade gestacional, avaliar junto à equipe a necessidade e a possibilidade de realizar corticoterapia^(26)^ 16. Planejar a cesárea em tempo hábil^(18,19,21,26)^ 17. Utilizar as habilidades de comunicação durante a assistência: tranquilizar a mulher; acolher de maneira empática e trabalhar a escuta ativa; manter sempre contato visual durante a comunicação; e, caso esteja consciente, informá-la sobre o quadro e permitir que tome decisões^(23,27)^ 18. Promover assistência com equipe multidisciplinar capacitada^(21)^, incluindo capacitação em casos de parada cardiorrespiratória^(18,21)^
**Puerpério imediato e mediato**
1. Avaliar/monitorar rigorosamente a loquiação de 15 em 15 minutos^(26)^, devido ao risco aumentado de sangramento 2. Após estabilização materna, promover vínculo com neonato^(26)^ 3. Assim que a puérpera estiver estável, estimular a amamentação ou realizar ordenha mecânica, se necessário^(26)^ 4. Observar a incisão cirúrgica quanto a sinais flogísticos e sangramentos^(24)^ 5. Promover cuidados assépticos na manipulação de sondas, drenos e cateteres^(24)^ 6. Promover cuidados de hidratação com a pele e realizar mudança de decúbito periodicamente, se necessário^(24)^ 7. Avaliar condições maternas e orientá-la quanto aos cuidados com neonato^(24)^
**Cuidados com familiares**
1. Garantir à família que mulher e feto estão recebendo o melhor cuidado possível^(23)^ 2. Respeitar a privacidade de cada membro da família^(23)^ 3. Escutar ativamente preocupações, questionamentos e sentimentos dos familiares^(23)^ 4. Fornecer informações sobre os cuidados de enfermagem realizados^(23)^ 5. Promover o envolvimento familiar nas decisões e cuidados^(23)^ 6. Oferecer suporte/apoio necessários^(23)^
**Cuidados na alta hospitalar ou na sua vigência**
1. Promover apoio emocional contínuo no período puerperal^(27)^ 2. Promover cuidado emocional/psicológico para puérperas e familiares em casos de perdas fetais/neonatais^(22,27)^ 3. Encaminhar na alta para grupo de apoio para partilhar e falar sobre sua experiência e poder ressignificá-la^(27)^

## DISCUSSÃO

Na presente revisão, foram mais frequentes diagnósticos de síndrome HELLP em mulheres na segunda década de vida, semelhante a outros estudos de caso e estudos populacionais^(28–31)^.

Semelhantemente, estudo realizado em Alagoas apontou predomínio de multíparas nos casos identificados^(30)^. Já estudo na Índia apontou maior frequência em primíparas^(29)^.

Quanto à idade gestacional do diagnóstico, observaram-se casos mais comumente no terceiro trimestre gestacional, seguidos por escassos casos no segundo trimestre^(32)^, semelhantemente aos estudos revisados e outros estudos^(28,29,31,33)^. Ressalta-se a importância dos cuidados de enfermagem no pré-natal, a fim de identificar os sinais e intervir precocemente, uma vez que estudo realizado em Alagoas apontou que em todos os casos as mulheres realizaram consultas pré-natais, sendo que 66% realizaram mais de seis consultas, conforme recomendação do Ministério da Saúde, e 44% realizaram consultas, contudo em número inferior ao recomendado^(30)^.

Estudo apontou a associação entre a síndrome HELLP e a síndrome do anticorpo antifosfolípide (SAF), e, nesses casos, aumento de nascimentos prematuros e mortes fetais. Assim, recomenda-se que, na suspeita de uma ou outra condição, sejam feitos o rastreio e o tratamento. O tratamento para SAF melhora o prognóstico neonatal, ao evitar ou melhorar a insuficiência placentária^(32)^.

Vale ressaltar a importância de rastreio dos sintomas, a partir da consulta de enfermagem, quer seja durante o pré-natal ou na admissão da parturiente. A dor abdominal súbita, abrupta e forte intensidade no quadrante superior direito, foi um sintoma descrito por 30%^(34)^ a 78% das mulheres^(35)^. Já o aumento pressórico esteve presente entre 65%^(35)^e 85%^(34)^ das mulheres. Alterações visuais foram descritas por 20%^(34)^ a 25% das mulheres^(29)^.

Em todos os estudos revisados, realizou-se cesárea de emergência, semelhante aos desfechos apresentados em outros estudos, cuja frequência variou de 88%^(30)^ a 91,1%^(31)^. Apenas estudo realizado na Índia apontou que 84% dos casos evoluíram para parto vaginal^(29)^. Ressalta-se que ultimar o parto de forma imediata e o mais rápida possível contribui para o melhor prognóstico materno e neonatal, sendo indicado como tratamento para os casos, o que contribui para o aumento das cesáreas de emergência neste caso^(36)^.

Indica-se o retardo oportuno do parto, caso a idade gestacional esteja entre 24 e 34 semanas e não haja comprometimento materno e fetal, a fim de realizar a corticoterapia para permitir a maturação pulmonar fetal^(36)^.

Entre os estudos revisados, não houve casos de óbitos neonatais, no entanto nota-se que é um desfecho comum em quadros graves^(28–31)^. Metanálise apontou que a síndrome HELLP aumenta o risco em 1,56 vezes de ter como desfecho natimortos^(37)^.

Entre os nascidos vivos, destaca-se aumento de casos de prematuridade^(29–31)^, com índices entre 46%^(29)^ e 67%^(31)^ dos nascimentos, aumento dos índices de Apgar inferiores a 7^(29,30)^ e aumento dos casos de restrição de crescimento intra-uterino e baixo peso ao nascimento^(29–31)^. Nesse sentido, são necessários cuidados de enfermagem para evitar a prematuridade e, quando não possível, oferecer assistência de enfermagem qualificada e prever a internação desses neonatos em unidades especializadas, a fim de se obter melhores desfechos.

Destaca-se ainda que metanálise apontou que a síndrome HELLP contribuiu para aumento do risco de insuficiência renal aguda em 4,87 vezes^(37)^ e em 3,7 vezes o risco de óbito materno^(37)^. Entre os desfechos dos casos, observaram-se internação em Unidade de Terapia Intensiva^(28,33)^ para monitorização, politransfusões^(28,29,33)^ e necessidade de transplante hepático^(33)^. Devido à criticidade, aponta-se a necessidade de monitorização rigorosa e cuidados intensivos após o parto^(32)^, para garantir melhor desfecho materno.

Destaca-se a necessidade de estender os cuidados para além do tratamento da síndrome, para preservação da saúde mental das mulheres, que pode ser abalada devido à gravidade/criticidade e possíveis perdas. Estudo de revisão apontou alterações de saúde mental em mulheres que tiveram síndrome HELLP, com destaque para aumento da prevalência de depressão, ansiedade e síndrome do estresse pós-traumático, no entanto, devido aos estudos não controlar fatores de confusão, os resultados foram inconclusivos^(38)^. Assim, cabem cuidados relativos à preservação ou restauração da saúde mental dessas mulheres, como citado nos artigos revisados.

Como descrito anteriormente, a síndrome HELLP é uma condição grave que, caso não tratada, pode evoluir para óbito materno. Assim, ao se pensar a assistência, é necessário pensar os possíveis atrasos capazes de impedir a mulher de receber cuidados adequados e necessários. Baseado no modelo teórico dos três atrasos, é necessário repensar a assistência nos três componentes. O primeiro atraso se dá na decisão em procurar o atendimento. Esse primeiro pilar é influenciado pela recusa da mulher e dos familiares^(39)^. Neste sentido, o fortalecimento das práticas educativas e a assistência pré-natal qualificada podem ser fundamentais para evitar o mesmo.

O segundo atraso refere-se ao percurso e ao alcance da mulher aos serviços de saúde, que englobam desde fatores geográficos até transporte e infraestrutura. Já o terceiro atraso consiste em receber cuidados adequados quando há acesso aos serviços de saúde^(39)^. Nesses pontos, é necessário garantir o acesso à saúde em rede, com seus diferentes níveis de atenção. Reforça-se a importância de os serviços de saúde pensarem o fenômeno dos atrasos para analisar soluções para a morbimortalidade materna.

Além disso, é preciso ponderar sobre os nós críticos da assistência. O estudo propõe um plano de cuidados baseado nas evidências, contudo identificamos a relevância: do fortalecimento da assistência na porta de entrada das maternidades, reforçando a metodologia do acolhimento e a classificação de risco como estratégia de qualificação; da garantia de dimensionamento de pessoal adequado, de forma que a presença da enfermeira cuidando de uma mulher com HELLP não inviabilize o cuidado pelas enfermeiras na assistência a outras parturientes; e do fortalecimento das ações de educação permanente com vistas ao cuidado compartilhado interprofissional qualificado.

Ressalta-se a importância da taxonomias para a tomada de decisão do enfermeiro^(40)^. Embora apenas dois estudos da presente revisão descreveram diagnósticos e intervenções e que não há para o quadro diagnósticos ou intervenções específicas, as taxonomias contribuem para o planejamento do cuidado^(24)^, assim como na tomada de decisões efetiva e eficaz diante de uma emergência obstétrica^(22)^, como é o caso da síndrome HELLP.

Contudo, nota-se escassez de estudos que abordem cuidados de enfermagem nos casos de síndrome HELLP, o que compromete a discussão dos achados e consiste em uma limitação deste estudo. Além disso, há poucos estudos publicados sobre o tema na atualidade, o que compromete a comparabilidade dos resultados. Ao mesmo tempo, aponta-se a implicação desta revisão para a prática, uma vez que foram analisados e são apresentados cuidados inerentes à cada fase do ciclo gravídico-puerperal em que se detecta a síndrome.

## CONCLUSÃO

A presente revisão apontou a magnitude da síndrome, contudo houve escassez de estudos. Observou-se predominância de estudos de caso, seguidos de estudos descritivos. A revisão possibilitou identificar 39 cuidados de enfermagem voltados à assistência da mulher com síndrome HELLP.
